# SOS! Hydrogen Sulfide Enhances the Flavonoid Early Warning System in Rice Plants to Cope with Thiocyanate Pollution

**DOI:** 10.3390/toxics12080591

**Published:** 2024-08-14

**Authors:** Peng Tian, Yu-Xi Feng, Yan-Hong Li

**Affiliations:** 1 College of Environmental Science and Engineering, Guilin University of Technology, Guilin 541004, China; ttalways@glut.edu.cn; 2 Guangxi Key Laboratory of Environmental Pollution Control Theory and Technology, Guilin University of Technology, Guilin 541004, China; yu-xifeng@foxmail.com; 3 Guangdong-Hong Kong Joint Laboratory for Carbon Neutrality, Jiangmen Laboratory of Carbon Science and Technology, Jiangmen 529199, China; 4 Collaborative Innovation Center for Water Pollution Control and Water Safety in Karst Area, Guilin University of Technology, Guilin 541004, China

**Keywords:** flavonoids, thiocyanate, hydrogen sulfide, rice

## Abstract

The presence of thiocyanate (SCN^−^) in irrigation water has adverse effects on both plant growth and crop output. Hydrogen sulfide (H_2_S) is an important gaseous signaling molecule that can alleviate SCN^−^ stress. Flavonoids are secondary compounds produced by plants and are ubiquitous in the plant kingdom. They play important roles in several physiological and biochemical processes. To investigate the effect of exogenous H_2_S on the growth of early rice plants under SCN^−^ stress, we carried out a hydroponic experiment focusing on the interaction of exogenous H_2_S with flavonoids. In this study, a hydroponic experiment was performed to investigate the behavior of SCN^−^ when subjected to varying effective doses (EC_20_: 24.0 mg/L; EC_50_: 96.0 mg/L; and EC_75_: 300.0 mg/L). The findings indicated that the relative growth rate (*RGR*) of the plants treated with H_2_S + SCN^−^ was greater than that of the plants treated with SCN^−^ alone. Higher amounts of flavonoids were detected in the shoots than in the roots, with more variability in the shoots. The early warning level results showed that most of the flavonoids were present at levels I and II, while quercetin was present at level IV. Genetic expression variation factor (*GEVF*) analyses revealed an increase in the quantity of “promoter genes” with increasing SCN^−^ concentration in both rice tissues. Furthermore, administering external H_2_S while exposing rice tissues to SCN^−^ resulted in a considerable decrease in the levels of reactive oxygen species. This study provides novel insights into the regulation of flavonoid levels in rice plants by exogenous H_2_S, facilitating enhanced resistance to SCN^−^ stress and promoting sustainable agriculture.

## 1. Introduction

Thiocyanate (SCN^−^) is a negatively charged ion that is well complexed with compounds in two structures: –S–C≡N and –N=C=S [1]. It can be produced naturally during the process of detoxifying cyanide (CN^−^) or can act as a protective agent against the invasion of pathogens in living organisms [2]. Furthermore, SCN^−^ has been extensively employed as a crucial chemical precursor in several industrial applications, including pesticides, herbicides, metallurgy, electroplating, dyeing, and thiourea production [1]. In general, naturally existing SCN^−^ is typically found in small amounts; however, human industrial activities often introduce excessive amounts of SCN^−^, which cannot fully react [3]. As a result, a significant volume of wastewater containing SCN^−^ is released into the environment. This not only leads to the waste of chemical raw materials but also has a severe negative impact on the environment. A survey of wastewater discharge conducted by several chemical companies revealed that the coking sector generates the greatest quantity of SCN^−^ at the most concentrated levels, reaching 200–1000 mg/L [4,5]. Nevertheless, the release of wastewater containing SCN^−^ in China is not limited by applicable standards and regulations [6]. Consequently, untreated SCN^−^ infiltrates the natural environment via water bodies, posing a significant hazard to the viability of plants, animals, and human health [7]. Research has demonstrated that an excessive amount of SCN^−^ in the human body hinders the transfer of halides to the thyroid, stomach, and cornea [8]. The effect of SCN^−^ on the central nervous system leads to symptoms such as irritability, anxiety, hallucinations, psychosis, mania, delirium, and convulsions [9]. The exposure of fish to excessive SCN^−^ wastewater leads to the inhibition of several enzymes, including Mg-ATPase, resulting in sudden death syndrome in fish, which manifests as convulsions, loss of balance and buoyancy, extreme stiffness, and death [10]. Furthermore, an excessive concentration of SCN^−^ in plants is a substantial hazard to their growth and development. This encompasses the disturbance of the antioxidant system, leading to an overabundance of reactive oxygen species (ROS), the deterioration of photosynthetic pigments, and the obstruction of nutritional absorption [11,12]. Ultimately, these factors can lead to lower plant yields and compromised quality.

Plants release a diverse range of metabolites throughout their growth and development, and it is postulated that the plant kingdom harbors approximately one million metabolites [13]. The majority of these molecules are secondary metabolites, commonly known as specialized metabolites, that serve various physiological and biochemical functions, such as protecting against infections, attracting pollinators, promoting seed germination, and facilitating communication [14]. Through an extensive evolutionary process, these secondary metabolites have developed a range of adaptation mechanisms necessary for survival in dynamic situations, enabling plants to flourish [15]. Flavonoids are typically a group of important secondary metabolites stored in the vesicles of plant cells and include chalcones, flavonoids, flavonols, anthocyanins, proanthocyanidins, and others [16]. The metabolic pathways of flavonoids have been extensively studied in the fields of biochemistry and molecular biology. Studies have demonstrated that certain flavonoids play a significant role in the growth, development, and stress resistance of plants [17]. Flavonoids are pigments that are commonly found in plants and are responsible for the wide range of colors they produce. For example, anthocyanins produce shades of red, amber, sky blue, and amethyst, while chalcone results in golden tones. On the other hand, flavonols and flavonoids give rise to white and yellowish hues. Flavonoids function as plant antitoxins or antioxidants, and are capable of scavenging ROS as well as safeguarding plants against biotic and abiotic stressors such as ultraviolet radiation, chilling stress, microbial infections, and insect ingestion [18,19,20,21].

Hydrogen sulfide (H_2_S) is a non-colored gas with a pungent odor similar to that of rotten eggs. At first, it was thought to have acute toxic effects on living organisms [22]. Recent research has shown that H_2_S can control several processes in plants, such as seed germination, stomatal movement, root development, and photosynthesis [23,24]. Furthermore, owing to its lipophilicity, H_2_S can easily penetrate cell membrane lipid bilayers and serve as a gasotransmitter in the modulation of plant stress tolerance, encompassing both biotic and abiotic stresses [25]. Indeed, exogenous H_2_S may significantly alleviate the detrimental impacts of SCN^−^ stress on the morphological characteristics of rice roots and enhance the turnover of D1 proteins, thereby improving the CO_2_ fixation capacity of rice plants [26,27]. To obtain a deeper understanding of the impact of external H_2_S on alleviating SCN^−^ stress, we conducted transcriptome studies, which revealed that exogenous H_2_S plays a role in modifying secondary metabolites in rice plants under SCN^−^ stress [28]. Plant physiological processes and reactions to stress rely heavily on the balance of different metabolites [29,30]. It is in fact clear that H_2_S interacts with these metabolites at various stages of cellular signaling.

Indeed, some studies have demonstrated that exogenous H_2_S can regulate how plants react to flavonoids, which in turn affects how plants grow and develop. For instance, exogenous H_2_S enhanced flavonoid accumulation, reduced the levels of H_2_O_2_, O_2_^•−^, and MDA in bananas, and postponed fruit ripening as well as senescence [31]. Exogenous H_2_S enhanced the structure of flax roots by regulating the levels of free amino acids and promoting the biosynthesis of lignin as well as flavonoids via the phenylpropanoid pathway [32]. Applying external H_2_S to spinach plants exposed to chromium stress caused an increase in flavonoid concentrations, which in turn boosted the activity of various antioxidant enzymes, such as SOD, POD, CAT, and APX, thereby enhancing spinach stress tolerance [33]. Taken together, these findings demonstrate that external influences can disrupt coordinated and precise metabolic processes in plants; however, exogenous H_2_S can effectively control these processes to maintain coordination and stability in plant metabolism. Rice serves as a primary food source for nearly half of the world’s population, with a cultivation area covering approximately 165 million hectares, accounting for around 11% of global agricultural land [34]. Moreover, rice is also a perfect candidate for toxicity research, particularly in hydroponic tests conducted on a small scale [35]. Therefore, there is an urgent need to explore how secondary metabolites interact with external H_2_S in response to SCN^−^ stress in rice plants. Thus, the following goals are covered by this study: (1) to quantify the changes in flavonoids, H_2_O_2_, and O_2_^•−^ in rice plants under SCN^−^ stress with or without exogenous H_2_S supplementation; (2) to quantify the transcript levels of genes associated with flavonoid biosynthesis; and (3) to explore the key flavonoids that interact with exogenous H_2_S.

## 2. Methods and Materials

### 2.1. Plant Growth and Experimental Design

After the rice seeds (*Oryza sativa* L. XZX 45, sourced from HUNAAS) were soaked in purified water for 24 h, the inferior seeds were removed. Then, the seeds were evenly spread in plastic cups filled with sand, maintaining a thickness of approximately one centimeter and a distance of one centimeter from the rim of the cup. The prepared seeds were placed in an incubator that maintained a constant temperature of 25 ± 0.5 °C, a relative humidity of 60%, and a light intensity of 7000 lux [36]. During the sand culture process, deionized water was applied every 12 h. After seed germination, the plants were irrigated with a nutrient solution (modified 8692) every 12 h [7]. After 16 days of growth, the rice plants were in the seedling stage, and they were subsequently rinsed with purified water. Ten rice plants that exhibited good growth and similar heights were selected as the experimental group. The selected rice plants were placed in a nutrient solution for approximately 12 h to acclimate to the environment. Subsequently, a short-term exposure experiment was conducted with four replicates assigned to each treatment group. A large amount of ammonium thiocyanate is usually added in various industrial activities, so ammonium thiocyanate was chosen as the source of SCN^−^ in this study, and NaHS is widely used as the source of H_2_S under hydroponic conditions [28]. Two sets of treatments were carried out:

(1) SCN^−^ treatments: Rice plants were exposed to nutrient solutions containing various levels of SCN^−^ (0, 24, 96, and 300 mg SCN/L) for 3 days [26]. The 0 mg SCN/L treatment served as control 1, which did not receive SCN^−^ supplementation.

(2) H_2_S + SCN^−^ treatments: Six hours prior to the experiment, the rice plants were pretreated with a 100 μM NaHS solution, which acted as the source of H_2_S, as determined in our previous study [28]. The plants were subsequently treated with altered nutritional solutions that matched the previously stated SCN^−^ concentrations. As with the prior treatment, the 0 mg SCN/L treatment served as control 2, which included the use of NaHS but did not involve the administration of SCN^−^.

The concentrations of SCN^−^ used here correspond to three distinct effective concentrations (EC_20_, EC_50_, and EC_75_) that match the relative inhibition rates of rice plants (20, 50%, and 75%, respectively), as determined in our previous study [28]. To minimize water evaporation and prevent the growth of algae, aluminum foil was placed over each flask. The ammonium thiocyanate (NH_4_SCN) and sodium hydrogen sulfide (NaHS) used were analytically pure, and the reagents were purchased from Aladdin Chemical Company, Shanghai, China.

### 2.2. Measurement of Growth Parameters

As detailed by Ling et al. [36], the relative growth rate (*RGR*) of rice plants had a strong influence on this study, and in accordance with our previous study the following formula was used to assess the growth effects of SCN^−^ on rice:

(1) $$RGR=\frac{M_{\left(F\right)}-M_{\left(I\right)}}{M_{\left(F\right)}}\times 100$$

Here, *M*(*F*) and *M*(*I*) represent the final and initial weights of the rice plants, respectively, measured in grams.

### 2.3. Quantification of Flavonoids in the Rice Plants

After 3 days of rice plant stress, both the roots and shoots of the rice plants were collected and pulverized using liquid nitrogen. Powdered tissue samples were utilized for extracting, purifying, and analyzing flavonoids. The flavonoids were quantitatively analyzed by ultrahigh-performance liquid chromatography–tandem mass spectrometry (UPLC-MS/MS). The procedures for extraction, purification, and analysis were conducted following the methods outlined by Gao et al., with detailed steps in the Supporting Information S1 [37]. For every sample, three biological replicates were performed.

### 2.4. Quantification of ROS Levels in Rice Plants

The levels of ROS, which include H_2_O_2_ and O_2_^•−^, in the rice plants were measured using the methods outlined in our previous study [38]. Determination of H_2_O_2_: First, 0.2 g of rice tissue was accurately weighed and placed in 4.0 mL of trichloroacetic acid solution (0.1%) for grinding to a uniform slurry in an ice bath. It was centrifuged at 10,000 rpm at 4 °C for 5 min. One milliliter of the supernatant was added to 1.0 mL of sodium phosphate buffer (10 mM, pH 7.0) and 2.0 mL of KI solution (1.0 M). The solution was mixed thoroughly and incubated in the dark for 20 min, after which the absorbance was measured at a wavelength of 390 nm. A 0.1% trichloroacetic acid solution was used as a blank control in place of the supernatant from the plant sample grinding solution.

Determination of O_2_^•−^: First, 0.2 g of rice tissue was accurately weighed and placed in 4 mL of potassium phosphate buffer (65 mM, pH 7.8). The mixture was ground to a uniform slurry in an ice bath and then centrifuged at 10,000 rpm at 4 °C for 15 min. One milliliter of the supernatant was added to 1.0 mL of potassium phosphate buffer along with 0.1 mL of hydroxylamine hydrochloride solution (10 mM). After incubating for 1 h, 1.0 mL of p-aminobenzenesulfonic acid solution (58 mM) and 1.0 mL of α-naphthylamine solution (17 mM) were added. After a further 20 min of incubation, the absorbance was measured at 530 nm. For the leaf samples, 4.1 mL of chloroform (to remove chlorophyll interference) was added, and the samples were centrifuged at 10,000 rpm for 3 min. The aqueous phase was removed, and the absorbance was measured. For every sample, four biological replicates were performed.

### 2.5. Modeling of Positive/Negative Feedback Networks of Flavonoids in Rice Plants

#### 2.5.1. Evaluating Genetic Expression Variation Factors

In previous work, the transcriptomic profiles of rice seedlings were analyzed under different EC conditions with SCN^−^ and H_2_S + SCN^−^ treatments, and detailed experimental procedures can be found in the Supporting Information S2. BLAST-p searches were then conducted in the RAP-DB (http://rapdb.dna.affrc.go.jp (accessed on 1 July 2024)) databases to identify genes associated with the biosynthesis and metabolism of flavonoid compounds in rice. Finally, these genes were matched with the results determined in the transcriptome.

To measure the impact of exogenous H_2_S on the transcriptional regulation of certain genes by flavonoids, gene expression variation factors (*GEVFs*) were measured [39]. The *GEVF* calculations were based on the fold change in gene expression between SCN^−^ therapies and H_2_S + SCN^−^ treatments, as explained by the following equation:

(2) $$GEVFs=\frac{FC_{\left(H_{2}S+{\mathrm{SCN}}^{-}\right)}-FC_{\left({\mathrm{SCN}}^{-}\right)}}{FC_{\left({\mathrm{SCN}}^{-}\right)}}\times 100\%$$

Here, the fold variation in gene expression as a consequence of H_2_S + SCN^−^ treatments is denoted by *FC*_(_*_H2S_*_+*SCN−*)_, while the fold variation in gene expression as a result of SCN^−^ treatments is represented by *FC_(_*_SCN−)_. The threshold values for *GEVFs* were determined to be greater than 25% or less than 25%, corresponding to the up- or downregulation of genes, respectively.

#### 2.5.2. Evaluation of Flavonoid Variation Factors

To better understand the variations in flavonoids in rice plants during SCN^−^ stress in the presence of H_2_S, we used flavonoid variant factors (*FVFs*), which are similar to gene expression variant factors, as follows:

(3) $$FVFs=\frac{CFC_{\left(H_{2}S+{\mathrm{SCN}}^{-}\right)}-CFC_{\left({\mathrm{SCN}}^{-}\right)}}{CFC_{\left({\mathrm{SCN}}^{-}\right)}}\times 100\%$$

Here, the fold change in flavonoid content as a consequence of H_2_S + SCN^−^ treatment is denoted by *CFC_(__H2S+SCN−)_*, while the fold change in gene expression as a result of SCN^−^ treatment is represented by *CFC_(_*_SCN−)_. The calculated absolute value of the *FVFs* is divided into four warning response levels, I, II, III and IV, according to 0~20%, 20~50%, 50~75% and >75%, respectively.

### 2.6. Data Analysis

The results are expressed as the mean ± SD of biological replicates. Multiple Tukey’s range tests were conducted using SPSS 20.0 to assess significant differences (*p* < 0.01 or *p* < 0.05) between the control and treatment groups [40]. The notation “a” signifies significant differences between treatments and control 1 (*p* < 0.05), whereas “b” denotes significant differences among treatments at the same SCN^−^ exposure concentration (*p* < 0.05). The analysis of variance tables are shown in the Supporting Information Tables S1–S6.

## 3. Results

### 3.1. The Relative Growth Rate of Rice Plants

**Figure 1:**
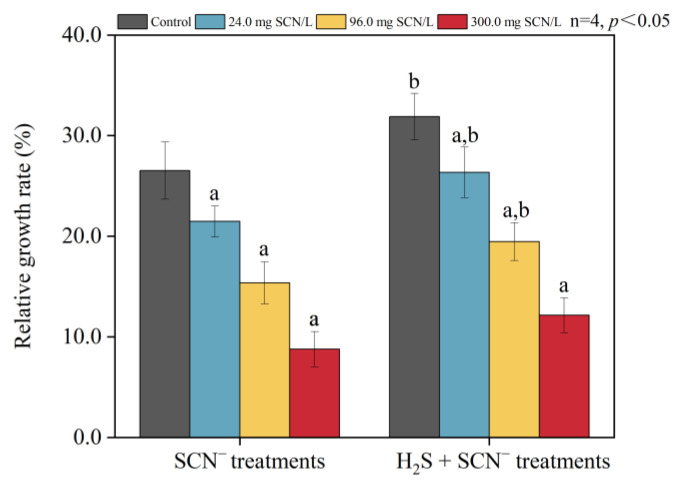
The relative growth rate of rice in the SCN^−^ treatments and H_2_S + SCN^−^ treatments.

The relative growth rate (*RGR*) of rice plants exposed to different concentrations of SCN^−^ significantly decreased in comparison to that of the control, as depicted in Figure 1. Similarly, rice plants treated with H_2_S exhibited a notable decrease in their *RGR* when subjected to varying concentrations of SCN^−^ (Figure 1). It is important to mention that the *RGR* of rice plants treated with exogenous H_2_S was greater than that of plants treated with exogenous H_2_S in response to only SCN^−^ stress.

### 3.2. Response of Rice Plants to Flavonoids

**Figure 2:**
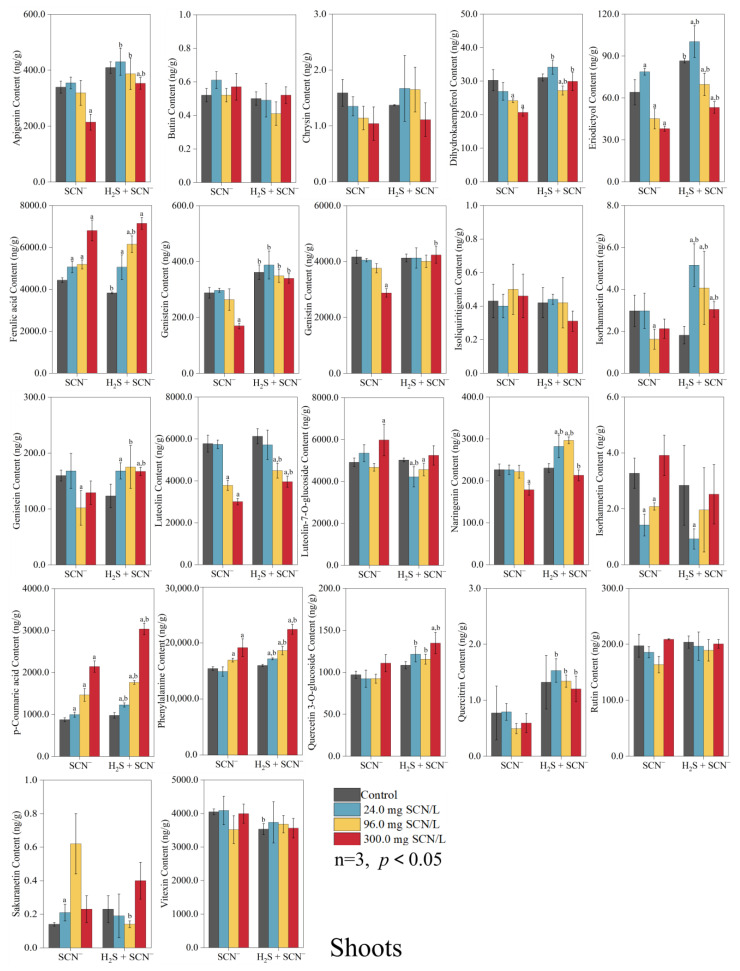
The content of flavonoids in shoots from the SCN^−^ treatments and H_2_S + SCN^−^ treatments.

**Figure 3:**
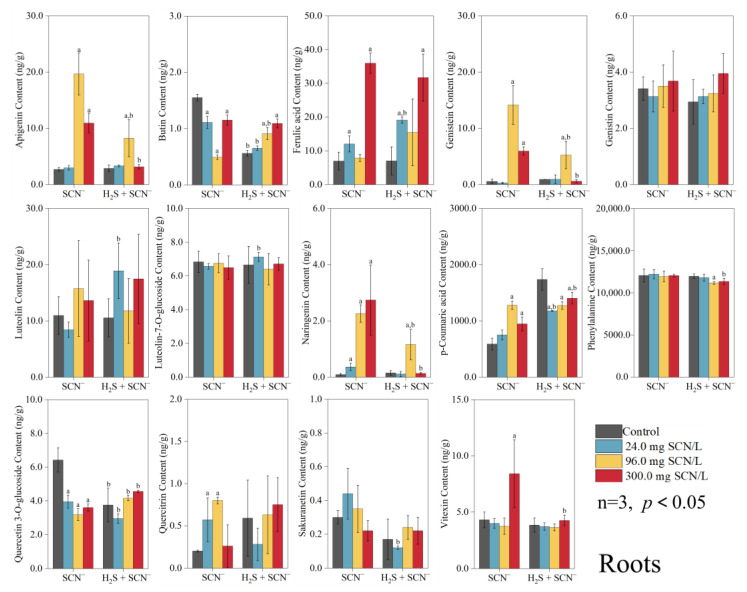
The content of flavonoids in roots from the SCN^−^ treatments and H_2_S + SCN^−^ treatments.

In the roots of the rice plants, 14 flavonoids were identified, whereas in the shoots, 22 flavonoids were detected. The changes in the levels of these compounds at different SCN^−^ concentrations are shown in Figure 2 and Figure 3.

In the roots of the rice plants at the EC_20_ level of SCN^−^, the contents of ferulic acid, luteolin, luteolin-7-O-glucoside, and p-coumaric acid under the H_2_S + SCN^−^ treatment were considerably greater (*p* < 0.05) than those under the SCN^−^ treatments, and the contents of butin, quercetin 3-O-glucoside, and sakuranetin were considerably lower (*p* < 0.05) than those under the SCN^−^ treatment. At the EC_50_ level of SCN^−^, butin, p-coumaric acid, and quercetin 3-O-glucoside in the H_2_S + SCN^−^ treatments group were considerably greater (*p* < 0.05) than those in the SCN^−^ treatment group, and the contents of apigenin, genistein, and naringenin were considerably lower (*p* < 0.05) than those in the SCN^−^ treatment group. Moreover, at the EC_75_, the levels of SCN^−^, p-coumaric acid, and quercetin 3-O-glucoside in the H_2_S + SCN^−^ treatment group were considerably greater (*p* < 0.05) than those in the SCN^−^ treatment group, and apigenin, genistein, naringenin, phenylalanine, and vitexin were considerably less abundant (*p* < 0.05) than those in the SCN^−^ treatment group.

In rice plant shoots at the EC_20_ level of SCN^−^, the contents of apigenin, dihydrokaempferol, eriodictyol, genistein, isoliquiritigenin, naringenin, p-coumaric acid, phenylalanine, quercetin 3-O-glucoside, and quercitrin under the H_2_S + SCN^−^ treatment were considerably greater (*p* < 0.05) than those under the SCN^−^ treatment, and only the levels of luteolin-7-O-glucoside were significantly lower (*p* < 0.05) than those under the SCN^−^ treatment. At the EC_50_ level of SCN^−^, increased contents of apigenin, dihydrokaempferol, eriodictyol, ferulic acid, genistein, isolioiritigenin, kaempferol, luteolin, naringenin, p-coumaric acid, phenylalanine, quercetin 3-O-glucoside, and quercitrin were detected in the H_2_S + SCN^−^ treatment group compared to the SCN^−^ treatment group (*p* < 0.05), and decreased levels of sakuranetin were detected compared to the SCN^−^ treatment group (*p* < 0.05). Finally, at the EC_75_ level of SCN^−^, apigenin, dihydrokaempferol, eriodictyol, genistein, genistin, isoliquiritigenin, kaempferol, luteolin, naringenin, p-coumaric acid, phenylalanine, quercetin 3-O-glucoside, and quercitrin were more abundant in the H_2_S + SCN^−^ treatment group than the SCN^−^ treatment group (*p* < 0.05).

### 3.3. ROS Levels in Rice Plants

**Figure 4:**
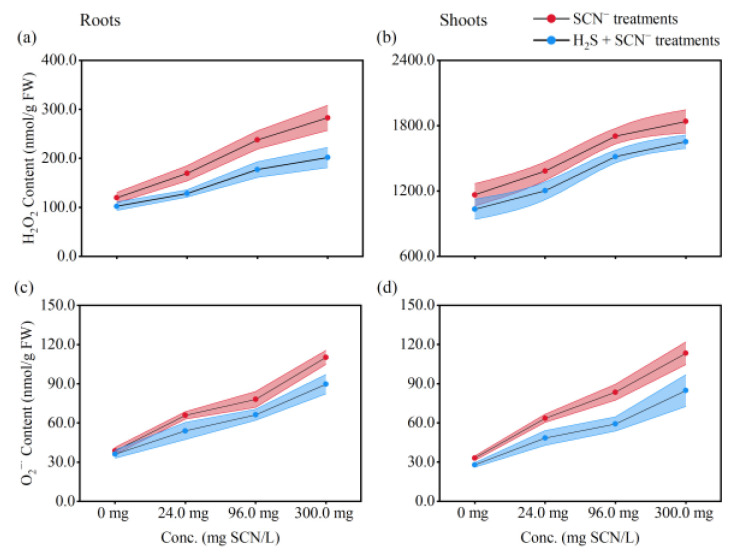
H_2_O_2_ and O_2_^•−^ content in rice seedlings from the SCN^−^ treatments and H_2_S + SCN^−^ treatments. (**a**): H_2_O_2_ content in roots. (**b**): H_2_O_2_ content in shoots. (**c**): O_2_^•−^ content in roots. (**d**): O_2_^•−^ content in shoots.

As depicted in Figure 4, when the concentration of SCN^−^ increased, a significant increase in O_2_^•−^ and H_2_O_2_ levels in the rice plants compared to those in the control plants was observed. Similarly, the inclusion of H_2_S resulted in a notable escalation in the production of O_2_^•−^ and H_2_O_2_ in the tissues of rice plants treated with SCN^−^, which exceeded that of control plants and increased with increasing SCN^−^ concentration. However, compared to the SCN^−^ treatment, the H_2_S + SCN^−^ treatment resulted in a lower level of ROS in the rice plants.

### 3.4. Evaluation of Flavonoid Early Warning Response Levels

**Figure 5:**
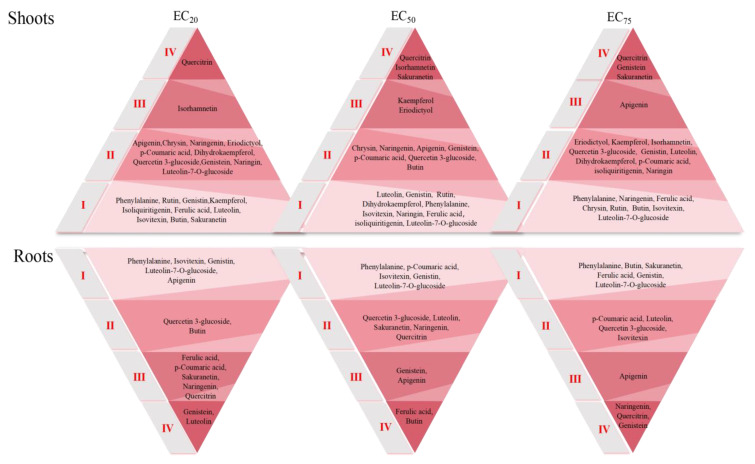
Early warning map of flavonoids in rice seedlings from the SCN^−^ treatments and H_2_S + SCN^−^ treatments.

The warning response level, derived from the calculations in Equation (2), is shown in Figure 5. Most of the flavonoids in the roots of the rice plants at the EC_20_ level of SCN^−^ were at the III level of warning, while most of the flavonoids in the shoots were at the II level of warning. At the EC_50_ level of SCN^−^, the quantity of flavonoids in both roots and shoots tissues of the rice plants was at warning levels I and II. Finally, at the EC_75_ level of SCN^−^, most of the flavonoids in the roots were at the I level of warning, while most of the flavonoids in the shoots were at the II level of warning. In addition, with an increase in SCN^−^ concentration, exogenous H_2_S changed the early warning level of several flavonoids, such as eriodictyol, kaempferol, and isorhamnetin. Quercetin in the shoots was always at the IV level of early warning under SCN^−^ stress, suggesting that SCN^−^ may be an important flavonoid regulated by exogenous H_2_S.

### 3.5. Response of Flavonoid-Biosynthesis- and Metabolism-Related Genes in Rice

Transcriptome results showed that a total of 37,872 rice genes were collected after eliminating redundant and non-informative data [28]. The total number of differentially expressed genes (DEGs) in rice seedlings was 1787, 7077, and 12,879 under SCN^−^ treatments at EC_20_, EC_50_, and EC_75_, respectively. In comparison, the number of DEGs under H_2_S + SCN^−^ treatments at the same EC levels (EC_20_, EC_50_, and EC_75_) was 4478, 8268, and 11,683, respectively [28]. GO and KEGG pathways showed that exogenous H_2_S triggered “secondary metabolite synthesis” in rice seedlings corresponding to different effective concentrations of SCN^−^ exposure [28].

**Figure 6:**
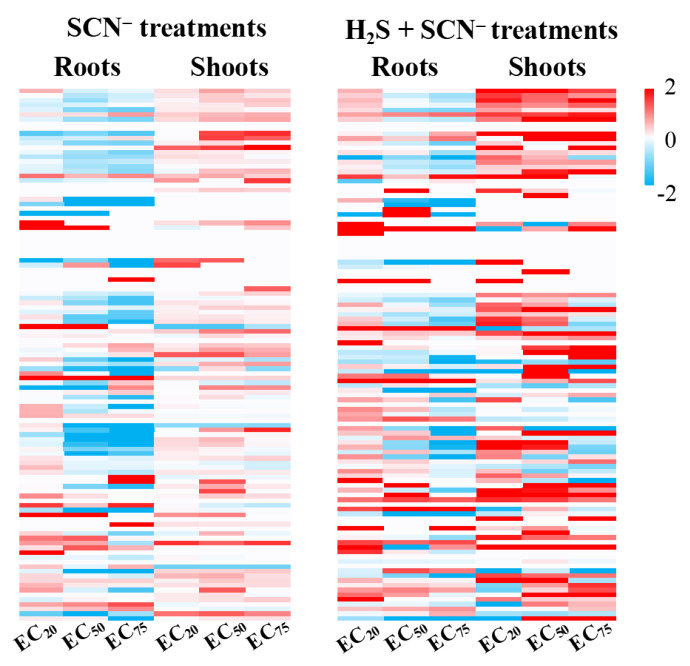
Heatmap of flavonoid-metabolism-related genes in rice seedlings under the SCN^−^ treatments and H_2_S + SCN^−^ treatments.

After screening and matching, a total of 113 genes related to flavonoid biosynthesis and metabolism in rice were identified, including PAL, C4H, 4CL, CHS, CHI, F3′H, F3H, FLS, etc. Detailed information on the relevant genes and transcriptomic results can be found in the Supporting Information Tables S7 and S8. Transcriptome analysis revealed that the expression levels of genes in the roots and shoots of rice seedlings varied under SCN^−^ stress at three different ECs. Following the application of H_2_S, most genes were upregulated. The heatmap (Figure 6) shows the changes in the expression of genes related to flavonoid metabolism in the roots and shoots of rice plants with and without exogenous H_2_S treatment. The results suggested a nuanced role of H_2_S in the response of rice seedlings to SCN^−^ stress and in the regulation of flavonoid metabolism.

### 3.6. Genetic Expression Variation Factors at Various EC Levels of SCN^−^

By applying Equation (1), we identified genes that either promoted (*GEVFs* > 25%) or repressed (*GEVFs* < 25%) flavonoid metabolism in rice plants, and the result was shown in Supporting Information Table S9. Analyses revealed an increase in the quantity of “promoter genes” in both rice tissues.

**Figure 7:**
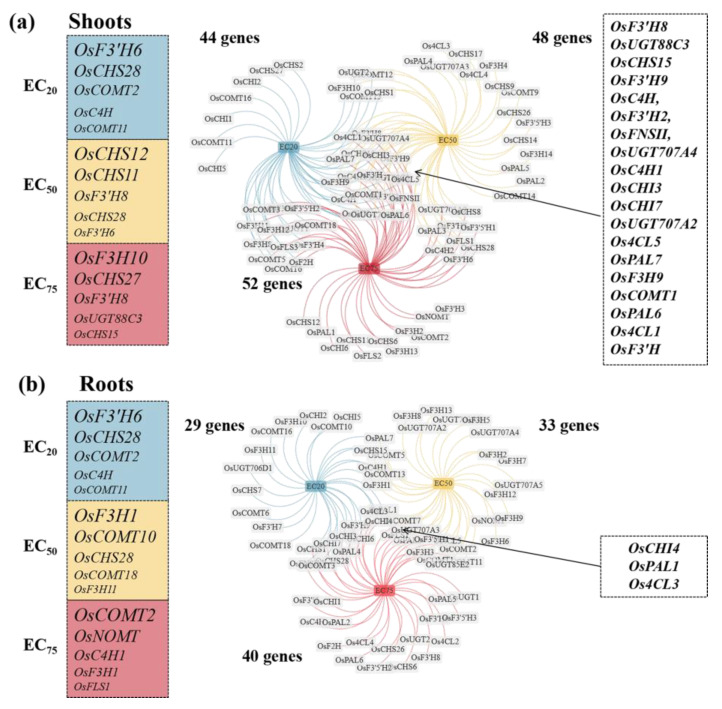
The genetic expression variation factors (*GEVFs*) between the SCN^−^ treatments and H_2_S + SCN^−^ treatments in rice seedlings. (**a**) Left: *GEVFs* of “promoting genes” in shoots at three ECs of SCN^−^ (a larger gene symbol indicates a greater number of *GEVFs*); right: Venn diagram of these “promoting genes” at three ECs of SCN^−^. (**b**) Left: *GEVFs* of “promoting genes” in root tissues at three ECs of the SCN^−^ (a larger gene symbol indicates a greater number of *GEVFs*); right: Venn diagram of these “promoting genes” at three ECs of the SCN^−^.

In rice seedling roots at the EC_20_ level of SCN^−^, *OsCHS28*, *OsF3H14*, *OsCHS26*, *OsF3H4*, and *OsUGT707A3* displayed greater *GEVFs*. At the EC_50_ level of SCN^−^, *OsCHS12*, *OsCHS11*, *OsF3′H8*, *OsCHS28,* and *OsF3′H6* displayed greater *GEVFs*. At the EC_75_ level of SCN^−^, *OsF3H10*, *OsCHS27*, *OsF3′H8*, *OsUGT88C3*, and *OsCHS15* exhibited greater amounts of *GEVFs*. A Venn diagram showing a total of 19 common “promoter genes” in roots is displayed in Figure 7. However, in rice seedling shoots at the EC_20_ level of SCN^−^, *OsF3′H6*, *OsCHS28*, *OsCOMT2*, *OsC4H*, and *OsCOMT11* displayed greater *GEVFs*. At the EC_50_ level of SCN^−^, *OsF3H1*, *OsCOMT10*, *OsCHS28*, *OsCOMT18*, and *OsF3H11* displayed greater *GEVFs*. Finally, at the EC_75_ level of SCN^−^, *OsCOMT2*, *OsNOMT*, *OsC4H1*, *OsF3H1*, and *OsFLS1* displayed greater *GEVFs*. A Venn diagram showed only three common “promoter genes” in the shoots (Figure 7).

## 4. Discussion

**Figure 8:**
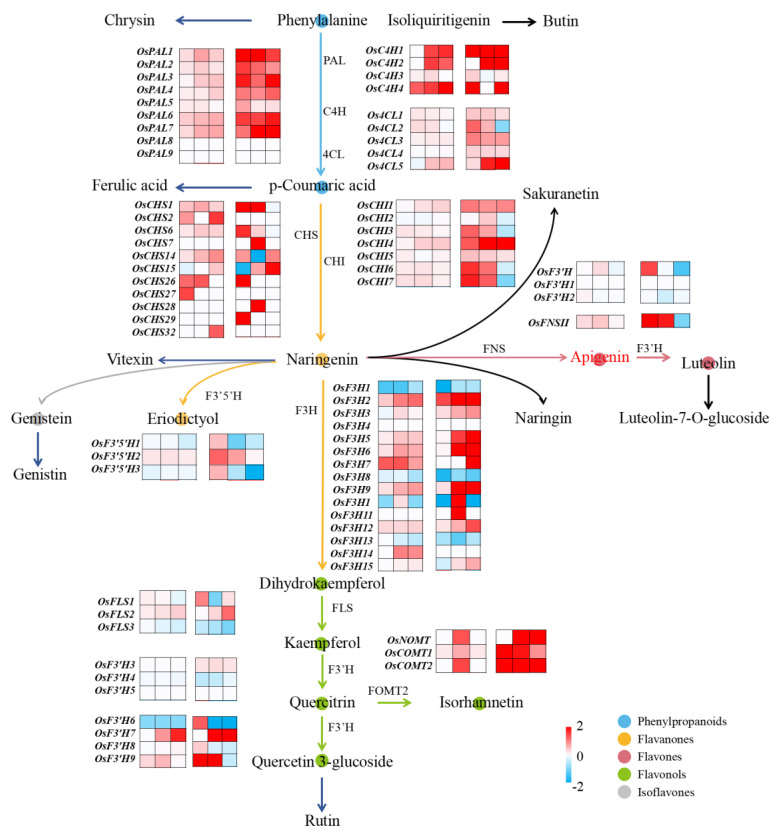
Expression pattern of flavonoid biosynthetic genes in rice shoots. The expression levels of the genes were expressed as log2 (fold change) values. (**Left**) SCN^−^ treatments. (**Right**) H_2_S + SCN^−^ treatments.

The growth and development of plants are intricately linked to the quantity of species and cellular metabolites present. This association underscores the critical synchronization between plant physiological processes and the abundance of specific metabolites within cells. Changes in metabolite levels and abnormalities in plant tissues play crucial roles in regulating signals and responding to stress in plants [41]. In the domain of toxicological assessments, the quantification of effective concentrations (ECs) has emerged as a pivotal tool, essential for evaluating the ecological risks posed by environmental contaminants and assessing their potential hazards [6,38,42]. Exposure to SCN^−^ can induce a diverse array of responses in plants, disrupting both physiological functions and molecular processes [7]. H_2_S, known as a plant growth regulator, is regarded as a beneficial “ameliorative agent” for mitigating the detrimental effects of SCN^−^ contamination on rice plants [26]. In the present work, we examined how the application of exogenous H_2_S influences both the resilience and endurance of rice plants to ECs caused by the presence of SCN^−^. This was achieved by reducing the accumulation of ROS through alterations in the levels of flavonoid compounds. Additionally, we analyzed the expression patterns of genes involved in flavonoid synthesis pathways in the leaves of rice plants (Figure 8). This work investigated the possible regulatory effects of externally applied H_2_S on biochemical and molecular processes in rice plants exposed to SCN^−^ stress.

The biosynthesis of flavonoids begins with the conversion of phenylalanine into various intermediate compounds through a series of enzymatic reactions and metabolic pathways [43]. Phenylalanine is converted into p-coumaroyl coenzyme A by the action of phenylalanine deaminase (PAL), cinnamic acid 4-hydroxylase (C4H), and 4-coumarate coenzyme A ligase (4CL). The enzyme phenylalanine ammonia-lyase (PAL) initiates the initial stage of the phenylalanine pathway by catalyzing the deamination of phenylalanine, resulting in the production of trans-cinnamic acid [44]. It has been reported that PAL activity can be induced by heavy metals, leading to enhanced phenolic acid accumulation [45]. In the general phenylpropanoid pathway, the second step is carried out by C4H, which is a cytochrome P450 monooxygenase found in plants. C4H catalyzes the hydroxylation of trans-cinnamic acid, converting it into p-coumaric acid. This reaction is the first oxidation reaction in the flavonoid production pathway [46]. The expression level of C4H in *Mauka poplar* and *Arabidopsis thaliana* was found to be directly related to the amount of lignin, a significant phenylpropanoid metabolite [43]. During the third stage, the enzyme 4CL facilitates the synthesis of p-coumaroyl coenzyme A by incorporating a coenzyme A (CoA) molecule into p-coumaric acid. The levels of flavonoids in plants are typically regulated by the coordinated expression of PAL, C4H, and 4CL [47]. The results of the present study showed that exogenous H_2_S treatment significantly increased the content of phenylalanine and p-coumaric acid in the shoots of rice plants with different EC levels of SCN^−^, with phenylalanine and p-coumaric acid at the primary and secondary alerts, respectively, while there were more *GEVFs* for the *OsPAL6*, *Os4CL1,* and *Os4CL5* genes in the transcriptome analysis. This suggests that the first step of exogenous H_2_S under SCN^−^ stress started to affect flavonoids.

Chalcone synthase (CHS) is a crucial enzyme in the initial stage of flavonoid production. It facilitates the synthesis of naringenin chalcone from p-coumaroyl coenzyme A. Chalcone isomerase (CHI) facilitates the rapid transformation of naringenin chalcone into naringenin, as well as the subsequent production of other flavonoids [48]. Naringenin, a naturally occurring antioxidant, may effectively remove ROS produced by stress by regulating the antioxidant metabolism in the chloroplasts of legumes, helping to mitigate the effects of short-term drought and salinity stress [49]. Enzyme genes are pivotal in the biosynthesis of flavonoids, with *AeCHS* identified as capable of augmenting flavonoid levels in *Arabidopsis thaliana* [50], and, in chrysanthemum, the upregulation of the CHI gene is associated with enhanced biosynthesis pathways, leading to the synthesis of lignans, quercetin, rutin, and apigenin [51]. Consistent with prior research, exogenous H_2_S under SCN^−^ stress notably elevated the content of naringenin in the shoots of rice plants in vivo under three different conditions of EC SCN^−^ concentration, and naringenin was involved in the II, II, and I responses; moreover, there were more *GEVFs* of regulated CHS and CHI genes, such as *OsCHS15*, *OsCHI3*, and *OsCHI7*, in the transcriptome. Therefore, we speculate that the increased naringenin may be used partly to resist SCN^−^ stress and partly to synthesize downstream flavonoids to further enhance the resistance of rice plants.

Furthermore, flavonoid biosynthesis, a crucial pathway in higher plants mediated by flavonoid synthase (FNS), involves the conversion of compounds, such as naringenin to apigenin, glycyrrhizin to dihydroxyflavonoids, pentosanol to luteolin, and pentahydroxyflavonoids to trichothecenes [51]. Here, two significantly upregulated flavonoids, apigenin and luteolin, were detected at three different EC SCN^−^ concentrations. Despite apigenin’s demonstrated ability to inhibit lipid peroxidation, scavenge ROS, bolster endogenous antioxidant defense mechanisms, and mitigate oxidative DNA damage in animal cells, its role in inducing responses to biotic or abiotic stresses in plants remains relatively underexplored in the existing literature [52]. Luteolin is a naturally occurring antioxidant that has a reduced antioxidant capacity compared to that of quercetin. However, it effectively scavenges ROS and provides protection to cells [53]. Salinity stress promotes the accumulation of lignans and their derivatives in *Lobelia*, *Japanese lotus*, *Lobelia*, *Lonicera*, and *Capsicum,* and enhances the defense of the plant antioxidant system [54]. The findings from this study demonstrated that, under SCN^−^ stress conditions, the application of exogenous H_2_S led to a significant increase in the levels of apigenin and lignans within the shoots of rice plants, the warning levels of apigenin were II, II, and III, and the warning levels of luteolin were I, I, and II at three different EC SCN^−^ concentrations. Thus, we speculated that both compounds also contribute to enhancing the resistance of rice plants to SCN^−^, with apigenin exerting a stronger influence than luteolin.

Flavonol synthase (FLS), a pivotal enzyme in flavonol biosynthesis, regulates the incorporation of flavonols into the branching pathway of flavonol synthesis, facilitating the production of diverse flavonol compounds, such as quercetin and kaempferol, through the action of flavonoid 3′-hydroxylase (F3′H) [55]. Under drought stress, both tea and pigeonpea exhibit substantial increases in the accumulation of flavonols, such as quercetin, and exhibit elevated expression levels of genes associated with flavonol synthesis, including CHI, F3′H, and FLS [56,57]. In this study, we observed a significant increase in the accumulation of flavonols, such as kaempferol, quercetin, isorhamnetin, and isoquercetin, in rice plants upon the application of exogenous H_2_S. Notably, these compounds exhibited a particularly robust response, with quercetin reaching a level IV warning level in terms of accumulation. Additionally, transcriptome analysis revealed that the *OsF3′H* gene had relatively high *GEVFs*.

Antioxidants are among the most important functions of flavonoids, and, in plants, flavonoids are often involved in oxidative stress response processes [58]. Oxidation is the process of transferring electrons from one atom to another and is an important part of the metabolism of aerobic life [59]; however, when the electron flow becomes uncoupled and free radicals are generated, the growth of an organism is adversely affected. Free radicals can initiate oxidative reactions by targeting lipids in cell membranes and proteins in tissues, which in turn induces oxidative reactions that can lead to membrane and tissue damage [60]. Flavonoids, as plant polyphenol species, are an important class of defensive antioxidants with potent free radical scavenging and antioxidant activities [58]. During this investigation, we observed that exposure to SCN^−^ resulted in a marked increase in oxidation levels in rice plants. Furthermore, our previous research underscored the influence of heightened reactive oxygen species levels on initiating processes such as lipid peroxidation, protein modification, and DNA damage in plant cells under SCN^−^ stress conditions [3]. The addition of exogenous H_2_S greatly decreased the levels of ROS in rice tissues that were exposed to SCN^−^. This highlights the ability of exogenous H_2_S to directly or indirectly mitigate the buildup of ROS in rice tissues under SCN^−^ stress. Based on these findings, we posited that exogenous H_2_S mitigates the ROS accumulation induced by SCN^−^ stress in rice plants through the modulation of flavonoid levels, thereby fostering rice plant growth.

## 5. Conclusions

This work explored the possible interactions between externally introduced H_2_S and flavonoids in the context of SCN^−^-induced stress. The exogenous application of H_2_S partially mitigated the rapid decline in growth indices observed in hydroponic rice plants treated with SCN^−^. A greater abundance of flavonoids was observed in the shoots of rice plants subjected to SCN^−^ stress, and exogenous H_2_S mainly regulated the flavonoid network in the leaves of rice plants. Genetic expression variation factor (*GEVF*) analyses revealed an increase in the abundance of “promoter genes” with increasing SCN^−^ concentration in both rice tissues, indicating that the greater the SCN^−^ concentration, the weaker the regulatory effect of exogenous H_2_S. Overall, the findings of this study establish a foundation for investigating the alterations in secondary metabolites in rice plants under SCN^−^ stress induced by exogenous H_2_S.

## Data Availability

Data will be available as demanded. All participants consented to the publishing of their data.

## Supplementary Materials

The following supporting information can be downloaded at: https://www.mdpi.com/article/10.3390/toxics12080591/s1, Table S1: The analysis of variance tables in roots at EC20 of SCN^−^. Table S2: The analysis of variance tables in roots at EC50 of SCN^−^. Table S3: The analysis of variance tables in roots at EC75 of SCN^−^. Table S4: The analysis of variance tables in shoots at EC20 of SCN^−^. Table S5: The analysis of variance tables in shoots at EC50 of SCN^−^. Table S6: The analysis of variance tables in shoots at EC75 of SCN^−^. Table S7: Transcriptome data of genes related to flavonoid synthesis under SCN^−^ treatments. Table S8: Transcriptome data of genes related to flavonoid synthesis under H2S + SCN^−^ treatments. Table S9: Then gene expression variation factors at different ECs of SCN^−^.
