# Supplementary material for: SOS! Hydrogen Sulfide Enhances the Flavonoid Early Warning System in Rice Plants to Cope with Thiocyanate Pollution
Source: Toxics. 2024 Aug 14;12(8):591. doi: 10.3390/toxics12080591 (PMC11359349; doi:10.3390/toxics12080591)
Supplement: Supplementary file 1 [file toxics-12-00591-s001.zip › toxics-3122499-supplementary.pdf]

Supporting information for

SOS! Hydrogen sulfide enhances the flavonoid early warning system in rice plants to cope with thiocyanate pollution

Peng Tian[1], Yu-Xi Feng[2,3], Yan-Hong Li[1,2,4*]

[1] College of Environmental Science and Engineering, Guilin University of Technology, Guilin 541004, China
[2] Guangxi Key Laboratory of Environmental Pollution Control Theory and Technology, Guilin University of Technology, Guilin 541004, China
[3] Guangdong-Hong Kong Joint Laboratory for Carbon Neutrality, Jiangmen Laboratory of Carbon Science and Technology, Jiangmen 529199, Guangdong Province, China
[4] Collaborative Innovation Center for Water Pollution Control and Water Safety in Karst Area, Guilin University of Technology, Guilin 541004, China

[*]Corresponding author
Prof. Dr. Yan-Hong Li
E-mail: lyh1685@163.com

Supporting Information S1.

Quantification of flavonoids in the rice plants. *Extraction and Purification of tissues samples*: Remove the sample from -80 ℃ storage, weigh 100 mg of the sample after grinding with liquid nitrogen. Add 300 μL extraction solution and 10 μL internal standard (10 μg/mL) to the sample, vortex thoroughly for 30 seconds, and sonicate in a water bath at 10°C for 0.5 hours at 14,000g. Centrifuge at 10 ℃ for 20 minutes at 14,000g, and transfer the supernatant to an Ostro 25 mg 96-well plate (Waters, 186005518) using a positive pressure device for filtration. Wash each well with 200 μL extraction solution once, then freeze at -80 ℃ for storage.

Conditions for HPLC: The mobile phase buffer included a combination of buffer A (0.1% formic acid in water) and buffer B (0.1% formic acid in acetonitrile). The injection volume was 2.0 μL at a flow rate of 400 μL/min, automatic sampler temperature was 4°C, and the column temperature was 45°C. The linear gradient was as follows: 5%→98% B (v/v) for 13 min; 98%→5% B (v/v) to 2 min.

Mass Spectrometry: The Sciex 5500 QTRAP mass spectrometer performs MRM (Multiple Reaction Monitoring) analysis in positive and negative ion switching modes. The ion source parameters for positive ion mode are as follows：Source temperature, 550 ℃, Gas 1, 55; Gas 2, 50; CRU, 30; ISVF, 5500 V. The ion source parameters for negative ion mode are as follows:550 ℃; Gas 1, 55; Gas 2, 50; CRU, 30; ISVF, -4500 V.

Supporting Information S2.

Transcriptome sequencing

Library preparation and Illumina HiSeq sequencing: The root and shoot tissues of rice seedlings treated by SCN^{-} and H_2S+SCN^{-} were subjected to RNA-Seq analysis. Each treatment included three biological replicates. RNA-seq transcriptome libraries were constructed using the TruSeq RNA Sample Preparation Kit (Illumina Inc., San Diego, CA, USA) in accordance with the manufacturer's guidelines. In brief, mRNA was isolated through poly(A) selection with oligo(dT) beads and subsequently fragmented using a fragmentation buffer. cDNA synthesis, end repair, A-tail addition, and ligation of Illumina-indexed adapters were carried out as per Illumina's protocol. The libraries were then size-selected to capture cDNA fragments within the range of 150–200 bp, followed by PCR amplification for 15 cycles using Phusion DNA polymerase (NEB). Following quantification with the TBS380, the libraries were sequenced on the Illumina NovaSeq 6000 platform (NovaSeq-PE 150) by Shanghai BIOZERON Co., Ltd.

Read quality control and mapping: Trimmomatic (Version 0.36; parameters: SLIDINGWIND OW: 4:15, MINLEN: 75) was employed to trim adapters, remove ambiguous bases, and eli minate low-quality raw reads. Subsequently, the cleaned reads were aligned to the rice refer ence genome (Os-Nipponbare-Reference-IRGSP-1.0, available at https://rapdb.dna.affrc.go.jp/do wnload/irgsp1.html) using HISAT2 software (Version 2.2.1) with default parameters and in orientation mode. The quality of the alignment data was assessed using Qualimap (Version 2.2.1). Gene read counts were determined using HTSeq (Version 0.11.1).

***Identification of differentially expressed genes (DEGs)*: The read counts for each gene w ere converted to FPKM (Fragments Per Kilobase of transcript per Million mapped rea ds) values using edgeR (version 4.2.0, available at http://www.bioconductor.org/packages/**

release/bioc/html/edgeR.html/) to facilitate comparison of gene expression levels. Differentially expressed genes (DEGs) were identified based on log2 fold change (log2FC) and false discovery rate (FDR). Statistical significance was determined with a p-value threshold of < 0.05. Then GO and KEGG functions of DEGs were analyzed.

Table S1 The analysis of variance tables in roots at EC_{20} of SCN^-.

		Df	SumSq	SqMean	F	P
Apigenin	group	1	0.1873	0.18727	2.035	0.227
	Residuals	4	0.3681	0.09202		
Butin	group	1	0.3174	0.3174	46.79	0.00239
	Residuals	4	0.0271	0.0068		
Ferulic_acid	group	1	74.91	74.91	22.78	0.00882
	Residuals	4	13.16	3.29		
Genistein	group	1	0.6273	0.6273	2.051	0.225
	Residuals	4	1.2235	0.3059		
Genistin	group	1	0.0001	0.00007	0	0.986
	Residuals	4	0.7323	0.18308		
Luteolin	group	1	164.8	164.85	12.61	0.0238
	Residuals	4	52.3	13.07		
Luteolin_7_O_glucoside	group	1	0.4648	0.4648	9.779	0.0353
	Residuals	4	0.1901	0.0475		
Naringenin	group	1	0.0938	0.09375	0.976	0.379
	Residuals	4	0.3843	0.09608		
p_Coumaric_acid	group	1	273745	273745	72.12	0.00105
	Residuals	4	15183	3796		
Phenylalanine	group	1	251310	251310	1.085	0.356
	Residuals	4	926188	231547		
Quercetin_3_O_glucoside	group	1	1.612	1.612	16.43	0.0154
	Residuals	4	0.3925	0.0981		
Quercitrin	group	1	0.1262	0.12615	2.479	0.19
	Residuals	4	0.2035	0.05088		

Sakuranetin	group	1	0.14415	0.14415	12.78	0.0233
	Residuals	4	0.04513	0.01128		
Vitexin	group	1	0.1176	0.1176	0.794	0.423
	Residuals	4	0.5924	0.1481		
H_2O_2	group	1	3370	3370	22.75	0.00309
	Residuals	6	889	148		
$O_2^{\cdot-}$	group	1	282.5	282.51	11.13	0.0157
	Residuals	6	152.2	25.37		

Table S2 The analysis of variance tables in roots at EC_{50} of SCN^{-}.

		Df	SumSq	SqMean	F	P
Apigenin	group	1	196.54	196.54	15.86	0.0164
	Residuals	4	49.56	12.39		
Butin	group	1	0.26042	0.26042	40.58	0.00311
	Residuals	4	0.02567	0.00642		
Ferulic_acid	group	1	86.79	86.79	1.745	0.257
	Residuals	4	198.96	49.74		
Genistein	group	1	119.08	119.08	13.4	0.0216
	Residuals	4	35.56	8.89		
Genistin	group	1	0.0963	0.0963	0.189	0.686
	Residuals	4	2.0401	0.51		
Luteolin	group	1	23.32	23.32	0.441	0.543
	Residuals	4	211.58	52.9		
Luteolin_7_O_glucoside	group	1	0.1803	0.1803	0.308	0.609
	Residuals	4	2.3435	0.5859		
Naringenin	group	1	2.483	2.483	1.053	0.363
	Residuals	4	9.434	2.359		
p_Coumaric_acid	group	1	56	56	0.011	0.921
	Residuals	4	20152	5038		
Phenylalanine	group	1	873833	873833	3.988	0.117
	Residuals	4	876528	219132		
Quercetin_3_O_glucoside	group	1	1.4603	1.4603	19.81	0.0112

	Residuals	4	0.2949	0.0737		
Quercitrin	group	1	0.0434	0.04335	0.397	0.563
	Residuals	4	0.4363	0.10908		
Sakuranetin	group	1	1.76	1.76	1.224	0.331
	Residuals	4	5.754	1.438		
Vitexin	group	1	0.015	0.015	0.049	0.836
	Residuals	4	1.224	0.3059		
H_2O_2	group	1	7346	7346	24.2	0.00266
	Residuals	6	1821	304		
$O_2^{\cdot -}$	group	1	7346	7346	24.2	0.00266
	Residuals	6	1821	304		

Table S3 The analysis of variance tables in roots at EC_{75} of SCN^{-}.

		Df	SumSq	SqMean	F	P
Apigenin	group	1	90.48	90.48	57.81	0.00161
	Residuals	4	6.26	1.57		
Butin	group	1	0.004817	0.004817	0.654	0.464
	Residuals	4	0.029467	0.007367		
Ferulic_acid	group	1	27.48	27.48	0.947	0.386
	Residuals	4	116.02	29.01		
Genistein	group	1	44.39	44.39	153.4	0.000244
	Residuals	4	1.16	0.29		
Genistin	group	1	0.099	0.0988	0.12	0.746
	Residuals	4	3.282	0.8206		
Luteolin	group	1	22.2	22.2	0.385	0.568
	Residuals	4	230.3	57.58		
Luteolin_7_O_glucoside	group	1	0.0704	0.07042	0.231	0.656
	Residuals	4	1.2181	0.30452		
Naringenin	group	1	10.192	10.192	13.01	0.0226
	Residuals	4	3.134	0.784		
p_Coumaric_acid	group	1	312739	312739	24.05	0.00802
	Residuals	4	52016	13004		

Phenylalanine	group	1	746560	746560	9.48	0.037
	Residuals	4	315010	78752		
Quercetin_3_O_glucoside	group	1	0.6667	0.6667	4.413	0.104
	Residuals	4	0.6043	0.1511		
Quercitrin	group	1	0.3504	0.3504	4.28	0.107
	Residuals	4	0.3275	0.0819		
Sakuranetin	group	1	0.138	0.138	0.972	0.38
	Residuals	4	0.5679	0.142		
Vitexin	group	1	25.92	25.917	5.524	0.0785
	Residuals	4	18.77	4.692		
H_2O_2	group	1	13148	13148	24.38	0.00261
	Residuals	6	3235	539		
$O_2^{\cdot-}$	group	1	13148	13148	24.38	0.00261
	Residuals	6	3235	539		

Table S4 The analysis of variance tables in shoots at EC_{20} of SCN^{-}.

		Df	SumSq	SqMean	F	P
Apigenin	group	1	8577	8577	6.164	0.068
	Residuals	4	5566	1391		
Butin	group	1	0.01927	0.019267	3.091	0.154
	Residuals	4	0.02493	0.006233		
Chrysin	group	1	0.1568	0.1568	0.829	0.414
	Residuals	4	0.7567	0.1892		
Dihydrokaempferol	group	1	78.7	78.7	13.66	0.0209
	Residuals	4	23.04	5.76		
Eriodictyol	group	1	699.8	699.8	10.29	0.0326
	Residuals	4	271.9	68		
Ferulic_acid	group	1	129	129	0.001	0.981
	Residuals	4	832816	208204		
Genistein	group	1	12294	12294	9.605	0.0362
	Residuals	4	5120	1280		
Genistin	group	1	9114	9114	0.136	0.731
	Residuals	4	267717	66929		

Isoliquiritigenin	group	1	0.002817	0.002817	0.983	0.378
	Residuals	4	0.011467	0.002867		
Isorhamnetin	group	1	7.107	7.107	8.095	0.0466
	Residuals	4	3.512	0.878		
Kaempferol	group	1	0	0	0	0.997
	Residuals	4	2377	594.3		
Luteolin	group	1	0	0	0	0.997
	Residuals	4	2377	594.3		
Luteolin_7_O_glucoside	group	1	1922238	1922238	9.816	0.0351
	Residuals	4	783325	195831		
Naringenin	group	1	4648	4648	11.14	0.0289
	Residuals	4	1669	417		
Naringin	group	1	0.375	0.375	2.634	0.18
	Residuals	4	0.5695	0.1424		
p_Coumaric_acid	group	1	80201	80201	25.83	0.00707
	Residuals	4	12421	3105		
Phenylalanine	group	1	7686054	7686054	20.92	0.0102
	Residuals	4	1469378	367344		
Quercetin_3_O_glucoside	group	1	1284.8	1284.8	13.59	0.0211
	Residuals	4	378.3	94.6		
Quercitrin	group	1	0.8214	0.8214	24.06	0.00801
	Residuals	4	0.1365	0.0341		
Rutin	group	1	174.7	174.7	0.473	0.529
	Residuals	4	1477.2	369.3		
Sakuranetin	group	1	0.00082	0.000817	0.085	0.785
	Residuals	4	0.03847	0.009617		
Vitexin	group	1	187748	187748	0.676	0.457
	Residuals	4	1111548	277887		
H_2O_2	group	1	65622	65622	9.271	0.0227
	Residuals	6	42470	7078		

$O_2^{\cdot-}$	group	1	451.7	451.7	21.52	0.00354
	Residuals	6	125.9	21		

Table S5 The analysis of variance tables in shoots at EC_{50} of SCN^{-}.

		Df	SumSq	SqMean	F	P
Apigenin	group	1	102	102	0.005	0.945
	Residuals	4	75892	18973		
Butin	group	1	0.01815	0.01815	5.672	0.0759
	Residuals	4	0.0128	0.0032		
Chrysin	group	1	0.3851	0.3851	3.745	0.125
	Residuals	4	0.4113	0.1028		
Dihydrokaempferol	group	1	13.741	13.741	13.23	0.022
	Residuals	4	4.156	1.039		
Eriodictyol	group	1	899.9	899.9	15.55	0.0169
	Residuals	4	231.5	57.9		
Ferulic_acid	group	1	1392855	1392855	13.95	0.0202
	Residuals	4	399463	99866		
Genistein	group	1	10837	10837	10.65	0.031
	Residuals	4	4070	1018		
Genistin	group	1	343769	343769	3.08	0.154
	Residuals	4	446428	111607		
Isoliquiritigenin	group	1	0.0096	0.0096	0.459	0.535
	Residuals	4	0.08373	0.02093		
Isorhamnetin	group	1	8.93	8.93	5.473	0.0794
	Residuals	4	6.526	1.632		
Kaempferol	group	1	8031	8031	6.586	0.0622
	Residuals	4	4878	1219		
Luteolin	group	1	747598	747598	8.364	0.0445
	Residuals	4	357546	89387		
Luteolin_7_O_glucoside	group	1	16766	16766	0.278	0.626
	Residuals	4	241578	60394		
Naringenin	group	1	8417	8417	57.95	0.0016
	Residuals	4	581	145		

Naringin	group	1	0.023	0.0228	0.02	0.895
	Residuals	4	4.607	1.1517		
p_Coumaric_acid	group	1	134087	134087	10.61	0.0312
	Residuals	4	50558	12639		
Phenylalanine	group	1	4324534	4324534	12.89	0.0229
	Residuals	4	1341561	335390		
Quercetin_3_O_glucoside	group	1	813.4	813.4	26.22	0.00688
	Residuals	4	124.1	31		
Quercitrin	group	1	0.4648	0.4648	3.533	0.133
	Residuals	4	0.5263	0.1316		
Rutin	group	1	976.1	976.1	3.325	0.142
	Residuals	4	1174.4	293.6		
Sakuranetin	group	1	0.3361	0.3361	19.39	0.0117
	Residuals	4	0.0693	0.0173		
Vitexin	group	1	39585	39585	0.331	0.596
	Residuals	4	477922	119481		
H_2O_2	group	1	70705	70705	16.55	0.00659
	Residuals	6	25638	4273		
$O_2^{\cdot-}$	group	1	1178.3	1178.3	35.34	0.00101
	Residuals	6	200.1	33.3		

Table S6 The analysis of variance tables in shoots at EC_{75} of SCN^{-}.

		Df	SumSq	SqMean	F	P
Apigenin	group	1	28851	28851	46.2	0.00245
	Residuals	4	2498	625		
Butin	group	1	0.00375	0.00375	0.801	0.421
	Residuals	4	0.01873	0.004683		
Chrysin	group	1	0.0088	0.00882	0.1	0.767
	Residuals	4	0.3517	0.08792		
Dihydrokaempferol	group	1	127.79	127.79	28.22	0.00604
	Residuals	4	18.11	4.53		
Eriodictyol	group	1	344	344	34.17	0.00427

	Residuals	4	40.3	10.1		
Ferulic_acid	group	1	177098	177098	1.094	0.355
	Residuals	4	647489	161872		
Genistein	group	1	43357	43357	221.7	0.000119
	Residuals	4	782	196		
Genistin	group	1	2300972	2300972	32.11	0.00478
	Residuals	4	286623	71656		
Isoliquiritigenin	group	1	0.03082	0.030817	3.15	0.151
	Residuals	4	0.03913	0.009783		
Isorhamnetin	group	1	1.1441	1.1441	8.132	0.0463
	Residuals	4	0.5627	0.1407		
Kaempferol	group	1	2171.3	2171.3	8.845	0.041
	Residuals	4	981.9	245.5		
Luteolin	group	1	1355708	1355708	29.52	0.00557
	Residuals	4	183668	45917		
Luteolin_7_O_glucoside	group	1	806285	806285	2.062	0.224
	Residuals	4	1564072	391018		
Naringenin	group	1	1799.9	1799.9	11.02	0.0294
	Residuals	4	653.2	163.3		
Naringin	group	1	2.912	2.912	3.543	0.133
	Residuals	4	3.288	0.822		
p_Coumaric_acid	group	1	1201269	1201269	65.04	0.00128
	Residuals	4	73877	18469		
Phenylalanine	group	1	16190780	16190780	9.919	0.0345
	Residuals	4	6529281	1632320		
Quercetin_3_O_glucoside	group	1	859	859	6.403	0.0646
	Residuals	4	536.6	134.2		
Quercitrin	group	1	0.5582	0.5582	13.52	0.0213
	Residuals	4	0.1652	0.0413		
Rutin	group	1	100.2	100.21	3.327	0.142
	Residuals	4	120.5	30.12		

Sakuranetin	group	1	0.04507	0.04507	5.112	0.0866
	Residuals	4	0.03527	0.00882		
Vitexin	group	1	280295	280295	3.37	0.14
	Residuals	4	332703	83176		
H_2O_2	group	1	69639	69639	9.252	0.0228
	Residuals	6	45162	7527		
$O_2^{\cdot -}$	group	1	1624.8	1624.8	14.56	0.00881
	Residuals	6	669.7	111.6		

Table S7 Transcriptome data of genes related to flavonoid synthesis under SCN^- treatments.

Gene	Locus ID	Roots			Shoots		
		EC_{20}	EC_{50}	EC_{75}	EC_{20}	EC_{50}	EC_{75}
OsPAL1	LOC_Os02g41630	1.83	0.74	0.86	2.43	11.98	3.82
OsPAL2	LOC_Os02g41650	1.00	0.55	0.69	1.89	3.71	1.96
OsPAL3	LOC_Os02g41670	0.79	0.43	0.74	1.27	4.11	4.93
OsPAL4	LOC_Os02g41680	0.69	0.77	1.01	1.52	1.66	3.70
OsPAL5	LOC_Os04g43760	0.79	0.38	0.31	1.65	1.82	1.17
OsPAL6	LOC_Os04g43800	1.28	1.30	2.23	3.81	6.48	8.80
OsPAL7	LOC_Os05g35290	0.80	0.43	0.35	2.60	8.57	10.98
OsPAL8	LOC_Os11g48110	1.00	1.00	1.00	1.00	1.00	1.00
OsPAL9	LOC_Os12g33610	1.00	1.00	1.00	1.00	1.00	1.00
OsC4H	LOC_Os02g26770	0.26	0.35	0.25	1.00	166.67	310.00
OsC4H1	LOC_Os02g26810	0.63	0.72	1.31	1.00	200.00	80.00
OsC4H2	LOC_Os05g25640	0.77	0.36	0.33	1.47	2.66	1.04
OsC4H3	LOC_Os01g60450	1.00	1.00	1.00	80.00	200.00	1280.00
Os4CL1	LOC_Os08g14760	0.89	0.49	0.34	1.88	1.64	1.23
Os4CL2	LOC_Os02g46970	0.56	0.44	0.44	2.54	2.12	0.87
Os4CL3	LOC_Os02g08100	0.93	0.54	0.61	1.46	1.73	1.60
Os4CL4	LOC_Os06g44620	0.81	0.50	0.41	0.99	1.16	1.12
Os4CL5	LOC_Os08g34790	0.79	0.59	0.39	0.69	5.37	6.93
OsCHS1	LOC_Os11g32650	3.21	1.79	2.58	5.00	10.33	3.21
OsCHS2	LOC_Os04g01354	0.63	0.79	0.87	16.67	1.00	220.00
OsCHS5	LOC_Os05g12190	1.00	1.00	1.00	1.00	1.00	1.00
OsCHS6	LOC_Os05g12210	1.00	1.00	1.00	1.86	4.04	3.25
OsCHS7	LOC_Os05g12240	1.00	1.00	1.00	1.00	1.00	1.00
OsCHS8	LOC_Os07g11440	1.23	0.07	0.00	1.00	1.00	1.00
OsCHS9	LOC_Os07g17010	0.35	0.01	0.01	1.00	1.00	1.00
OsCHS11	LOC_Os07g31750	1.00	1.00	1.00	1.00	1.00	1.00
OsCHS12	LOC_Os07g31770	0.04	0.04	1.00	1.00	1.00	1.00
OsCHS13	LOC_Os07g34140	1.00	1.00	1.00	1.00	1.00	1.00

OsCHS14	LOC_Os07g34190	16.67	1.00	1.00	2.19	6.76	22.24
OsCHS15	LOC_Os07g34260	33.33	73.33	1.00	0.48	1.02	4.33
OsCHS17	LOC_Os10g07616	1.00	1.00	1.00	1.00	1.00	1.00
OsCHS19	LOC_Os10g08670	1.00	1.00	1.00	1.00	1.00	1.00
OsCHS20	LOC_Os10g08710	1.00	1.00	1.00	1.00	1.00	1.00
OsCHS21	LOC_Os10g09860	1.00	1.00	1.00	1.00	1.00	1.00
OsCHS23	LOC_Os11g32540	1.00	1.00	1.00	1.00	1.00	1.00
OsCHS25	LOC_Os11g32610	1.00	1.00	1.00	1.00	1.00	1.00
OsCHS26	LOC_Os11g32620	0.01	0.34	0.01	56.67	93.33	1.00
OsCHS27	LOC_Os05g41645	0.69	2.35	0.01	156.67	1.00	1.00
OsCHS28	LOC_Os11g32540	1.00	1.00	1.00	1.00	1.00	1.00
OsCHS28	LOC_Os11g32540	1.00	1.00	1.00	1.00	1.00	1.00
OsCHS28	LOC_Os11g35930	1.00	1.00	23.33	1.00	1.00	1.00
OsCHS29	LOC_Os11g32580	1.00	1.00	1.00	1.00	1.00	1.00
OsCHS32	LOC_Os10g07040	1.00	1.00	1.00	1.00	1.00	80.00
OsCHI1	LOC_Os02g21520	0.95	0.71	0.64	1.16	2.20	3.17
OsCHI2	LOC_Os02g53810	0.66	0.52	0.23	0.86	0.74	1.09
OsCHI3	LOC_Os03g60509	1.18	0.37	0.22	1.96	1.64	1.19
OsCHI4	LOC_Os06g10210	0.97	1.10	0.80	1.33	3.45	4.07
OsCHI5	LOC_Os07g38390	0.79	0.75	0.28	1.51	0.90	1.38
OsCHI6	LOC_Os11g02440	1.25	0.26	0.27	1.72	2.00	1.41
OsCHI7	LOC_Os12g02370	0.88	0.30	0.15	2.25	2.59	1.45
OsF3H1	LOC_Os04g56700	43.33	16.67	1.00	0.01	0.01	0.11
OsF3H2	LOC_Os03g03034	1.04	0.94	1.25	3.98	31.89	59.50
OsF3H3	LOC_Os04g57160	1.09	1.16	1.00	0.65	2.64	1.29
OsF3H4	LOC_Os11g25060	1.00	1.00	1.00	1.00	1.00	1.00
OsF3H5	LOC_Os10g40934	1.02	1.35	2.06	1.57	4.50	4.27
OsF3H6	LOC_Os10g40934	0.89	1.00	1.78	2.13	13.23	8.56
OsF3H7	LOC_Os10g40900	0.95	0.84	1.23	90.00	110.00	13.33
OsF3H8	LOC_Os10g40880	1.47	0.29	0.00	0.43	0.51	0.11
OsF3H9	LOC_Os10g39140	1.01	0.61	0.48	2.15	8.49	14.35
OsF3H10	LOC_Os09g18450	0.45	0.17	0.00	0.04	2.29	0.04
OsF3H11	LOC_Os09g18390	2.60	0.90	0.12	1.00	1.00	1.00
OsF3H12	LOC_Os08g37456	173.33	23.33	140.00	2.72	2.80	3.16
OsF3H13	LOC_Os06g08032	0.85	0.37	1.56	0.97	0.37	0.19
OsF3H14	LOC_Os06g08023	0.02	0.02	2.63	1.00	30.00	23.33
OsF3H15	LOC_Os06g06720	1.00	1.15	1.50	1.04	1.43	1.69
OsF3'H	LOC_Os10g17260	0.98	0.49	0.17	1.06	2.50	0.63
OsF3'H1	LOC_Os01g50490	1.00	1.00	1.00	1.00	1.00	1.00
OsF3'H2	LOC_Os02g29960	1.86	0.65	0.11	1.34	0.85	0.93
OsF3'H3	LOC_Os08g35510	1.87	0.52	0.93	1.00	1.00	1.00
OsF3'H4	LOC_Os08g43440	1.90	1.32	1.31	0.78	0.86	0.73
OsF3'H5	LOC_Os09g08920	1.00	1.00	1.00	1.00	1.00	1.00
OsF3'H6	LOC_Os09g26970	1.22	0.02	0.02	0.04	0.04	0.04
OsF3'H7	LOC_Os09g26970	1.09	0.22	0.01	1.00	16.67	410.00
OsF3'H8	LOC_Os09g26980	0.45	0.03	0.03	0.93	1.14	1.25

OsF3'H9	LOC_Os10g16974	0.84	0.12	0.01	2.70	6.53	1.14
OsFLS1	LOC_Os02g52840	0.94	0.11	0.23	1.50	1.18	0.57
OsFLS3	LOC_Os10g41020	1.24	0.93	0.80	1.57	2.15	3.43
OsFLS2	LOC_Os10g40990	1.33	0.83	0.86	0.90	0.49	0.34
OsNOMT	LOC_Os12g13800	1.00	1.00	23.33	1.00	110.00	1.00
OsCOMT1	LOC_Os08g06100	1.04	0.34	0.18	1.72	9.04	1.80
OsCOMT2	LOC_Os09g17560	1.00	1.00	1.00	1.00	153.33	1.00
OsCOMT3	LOC_Os08g38910	2.57	1.29	1.02	1.25	3.90	2.59
OsCOMT4	LOC_Os12g25860	1.00	1.00	1.00	1.00	1.00	1.00
OsCOMT5	LOC_Os12g25820	5.00	1.56	6.00	1.09	0.36	0.17
OsCOMT6	LOC_Os12g25490	0.50	0.01	0.03	1.18	0.69	0.88
OsCOMT7	LOC_Os12g25450	13.33	53.33	1.00	20.00	30.00	1.00
OsCOMT8	LOC_Os12g10140	1.00	1.00	1.00	1.00	1.00	1.00
OsCOMT9	LOC_Os12g09770	1.00	1.00	26.67	2.17	1.91	1.84
OsCOMT10	LOC_Os11g33300	1.00	1.00	1.00	1.00	1.00	1.00
OsCOMT11	LOC_Os11g20160	1.40	0.72	0.33	0.50	7.58	0.58
OsCOMT12	LOC_Os11g20090	3.03	3.82	1.06	1.00	1.00	1.00
OsCOMT13	LOC_Os11g20080	2.29	2.00	0.50	276.67	76.67	233.33
OsCOMT14	LOC_Os11g20040	1.27	3.91	1.82	1.00	1.00	1.00
OsCOMT15	LOC_Os11g19880	16.67	1.00	1.00	1.00	1.00	1.00
OsCOMT16	LOC_Os02g57760	0.82	0.86	0.62	1.26	0.81	1.00
OsCOMT17	LOC_Os05g43930	1.00	1.00	1.00	1.00	1.00	1.00
OsCOMT18	LOC_Os05g43940	1.58	0.83	1.83	0.02	0.02	0.02
OsF3'5'H1	LOC_Os03g25150	1.73	0.83	0.83	0.80	0.82	0.33
OsF3'5'H2	LOC_Os03g25150	1.23	0.72	0.55	1.87	2.10	1.64
OsF3'5'H3	LOC_Os02g11700	1.00	1.00	23.33	0.54	0.77	0.68
OsUGT1	LOC_Os06g09240	0.80	0.03	0.00	2.98	3.16	4.23
OsUGT2	LOC_Os06g18670	1.36	0.91	0.73	6.82	8.73	2.38
OsUGT707A5	LOC_Os07g32010	1.37	1.44	1.46	1.44	4.14	2.60
OsUGT707A2	LOC_Os07g32060	1.81	1.19	1.12	2.24	2.19	2.57
OsUGT707A4	LOC_Os07g31960	2.17	0.73	0.23	1.00	86.67	1.00
OsUGT707A3	LOC_Os07g32020	1.00	1.00	1.00	1.73	2.27	0.89
OsUGT706D1	LOC_Os01g53460	0.75	1.10	1.02	1.73	2.27	3.80
OsUGT85E2	LOC_Os02g51910	2.07	2.61	4.19	0.63	1.04	1.12
OsUGT85A8	LOC_Os02g51900	1.26	1.70	2.53	0.92	0.60	0.51
OsUGT703A2	LOC_Os01g45110	0.29	0.01	0.46	46.57	84.86	27.29
OsUGT88C3	LOC_Os01g53370	1.17	1.13	0.01	1.09	1.76	2.10
OsFNSII	LOC_Os04g01140	0.79	0.18	0.04	2.57	4.22	1.23
OsF2H	LOC_Os06g01250	1.09	0.34	0.40	2.00	1.32	0.75

Table S8 Transcriptome data of genes related to flavonoid synthesis under H_2S + SCN^- treatments.

Gene	Locus ID	Roots			Shoots		
		EC_{20}	EC_{50}	EC_{75}	EC_{20}	EC_{50}	EC_{75}
OsPAL1	LOC_Os02g41630	1.84	0.97	1.07	11.06	16.27	4.85

OsPAL2	LOC_Os02g41650	1.46	0.65	0.46	4.91	3.47	2.42
OsPAL3	LOC_Os02g41670	1.76	0.92	0.56	6.48	3.37	7.80
OsPAL4	LOC_Os02g41680	1.51	0.96	0.86	2.93	2.54	3.61
OsPAL5	LOC_Os04g43760	1.33	0.37	0.35	2.10	1.20	1.29
OsPAL6	LOC_Os04g43800	2.12	2.68	3.26	4.92	4.63	6.58
OsPAL7	LOC_Os05g35290	1.48	0.66	0.58	3.06	11.18	26.30
OsPAL8	LOC_Os11g48110	1.00	1.00	1.00	1.00	1.00	1.00
OsPAL9	LOC_Os12g33610	1.00	1.00	1.00	1.00	1.00	1.00
OsC4H	LOC_Os02g26770	1.10	0.78	1.98	13.33	160.00	193.33
OsC4H1	LOC_Os02g26810	2.10	1.49	3.05	1.00	966.67	766.67
OsC4H2	LOC_Os05g25640	1.16	0.65	0.30	1.44	0.91	1.17
OsC4H3	LOC_Os01g60450	1.00	1.00	1.00	13.33	1.00	353.33
Os4CL1	LOC_Os08g14760	1.25	1.15	0.47	1.55	1.52	1.30
Os4CL2	LOC_Os02g46970	0.02	0.38	0.02	3.53	1.80	0.39
Os4CL3	LOC_Os02g08100	1.27	0.64	0.53	2.84	2.18	2.24
Os4CL4	LOC_Os06g44620	1.11	0.56	0.43	1.43	1.39	1.34
Os4CL5	LOC_Os08g34790	1.25	0.81	0.71	1.32	6.29	19.07
OsCHS1	LOC_Os11g32650	4.83	1.67	7.21	18.37	13.35	0.91
OsCHS2	LOC_Os04g01354	0.26	0.95	1.24	1.00	1.00	1.00
OsCHS5	LOC_Os05g12190	1.00	1.00	1.00	1.00	1.00	1.00
OsCHS6	LOC_Os05g12210	1.00	20.00	1.00	5.50	1.43	0.93
OsCHS7	LOC_Os05g12240	1.00	1.00	1.00	1.00	20.00	1.00
OsCHS8	LOC_Os07g11440	1.90	0.17	0.00	1.00	1.00	1.00
OsCHS9	LOC_Os07g17010	1.00	0.01	0.01	1.00	1.00	1.00
OsCHS11	LOC_Os07g31750	1.00	96.67	1.00	1.00	1.00	1.00
OsCHS12	LOC_Os07g31770	0.04	62.63	0.04	1.00	1.00	1.00
OsCHS13	LOC_Os07g34140	1.00	1.00	1.00	1.00	1.00	1.00
OsCHS14	LOC_Os07g34190	53.33	1.00	1.00	2.29	0.01	2.71
OsCHS15	LOC_Os07g34260	243.33	393.33	53.33	0.15	2.05	7.52
OsCHS17	LOC_Os10g07616	16.67	1.00	1.00	1.00	1.00	1.00
OsCHS19	LOC_Os10g08670	1.00	1.00	1.00	1.00	1.00	1.00
OsCHS20	LOC_Os10g08710	1.00	1.00	1.00	1.00	1.00	1.00
OsCHS21	LOC_Os10g09860	1.00	1.00	1.00	1.00	1.00	1.00
OsCHS23	LOC_Os11g32540	1.00	1.00	1.00	1.00	1.00	1.00
OsCHS25	LOC_Os11g32610	1.00	1.00	1.00	1.00	1.00	1.00
OsCHS26	LOC_Os11g32620	0.55	0.01	0.01	86.67	1.00	1.00
OsCHS27	LOC_Os05g41645	0.69	0.88	1.04	1.00	1.00	1.00
OsCHS28	LOC_Os11g32540	1.00	1.00	1.00	1.00	43.33	1.00
OsCHS28	LOC_Os11g32540	1.00	1.00	1.00	1.00	1.00	1.00
OsCHS28	LOC_Os11g35930	383.33	73.33	1.00	86.67	1.00	1.00
OsCHS29	LOC_Os11g32580	1.00	1.00	1.00	1.00	1.00	1.00
OsCHS32	LOC_Os10g07040	1.00	1.00	1.00	1.00	1.00	1.00
OsCHI1	LOC_Os02g21520	0.86	0.77	0.80	2.95	2.41	2.65
OsCHI2	LOC_Os02g53810	0.70	0.51	0.33	1.05	1.50	0.77
OsCHI3	LOC_Os03g60509	2.00	0.89	0.45	3.56	2.09	0.56
OsCHI4	LOC_Os06g10210	1.09	1.12	0.78	3.92	8.00	8.47

OsCHI5	LOC_Os07g38390	0.72	0.49	0.70	1.66	1.46	1.26
OsCHI6	LOC_Os11g02440	1.51	0.68	0.23	5.38	3.20	0.72
OsCHI7	LOC_Os12g02370	1.78	0.45	0.30	6.26	3.73	0.36
OsF3H1	LOC_Os04g56700	46.67	50.00	30.00	0.01	0.53	0.53
OsF3H2	LOC_Os03g03034	1.22	1.57	1.49	4.42	30.06	88.00
OsF3H3	LOC_Os04g57160	1.15	1.33	0.90	1.31	1.93	2.44
OsF3H4	LOC_Os11g25060	46.67	1.00	1.00	1.00	1.00	1.00
OsF3H5	LOC_Os10g40934	0.90	1.29	1.03	1.06	4.84	9.28
OsF3H6	LOC_Os10g40934	0.59	0.57	0.88	1.07	9.43	14.07
OsF3H7	LOC_Os10g40900	0.70	0.65	0.14	1.00	1.00	20.00
OsF3H8	LOC_Os10g40880	0.48	0.57	0.10	0.16	0.41	0.27
OsF3H9	LOC_Os10g39140	1.51	1.12	0.72	1.27	9.72	25.10
OsF3H10	LOC_Os09g18450	0.63	0.12	0.02	0.04	15.57	0.04
OsF3H11	LOC_Os09g18390	2.99	3.27	1.14	1.00	23.33	1.00
OsF3H12	LOC_Os08g37456	196.67	183.33	196.67	1.23	1.88	4.26
OsF3H13	LOC_Os06g08032	0.59	1.22	0.96	0.50	0.27	0.55
OsF3H14	LOC_Os06g08023	1.19	0.02	0.02	1.00	1.00	1.00
OsF3H15	LOC_Os06g06720	1.06	1.06	1.51	0.70	1.28	1.94
OsF3'H	LOC_Os10g17260	2.09	1.47	0.22	4.47	0.95	0.23
OsF3'H1	LOC_Os01g50490	1.00	1.00	1.00	1.00	1.00	1.00
OsF3'H2	LOC_Os02g29960	2.54	1.72	0.55	0.97	0.69	0.92
OsF3'H3	LOC_Os08g35510	2.03	0.90	0.66	1.00	1.00	1.00
OsF3'H4	LOC_Os08g43440	2.30	4.07	2.97	0.61	0.63	0.86
OsF3'H5	LOC_Os09g08920	1.00	1.00	1.00	1.00	1.00	1.00
OsF3'H6	LOC_Os09g26970	2.17	0.39	0.02	3.71	0.04	0.04
OsF3'H7	LOC_Os09g26970	1.61	1.52	0.01	1.00	163.33	30.00
OsF3'H8	LOC_Os09g26980	0.82	2.27	1.82	1.49	0.76	0.69
OsF3'H9	LOC_Os10g16974	1.21	0.40	0.12	15.71	9.01	0.64
OsFLS1	LOC_Os02g52840	2.43	0.32	0.00	2.72	0.34	1.27
OsFLS3	LOC_Os10g41020	1.20	1.46	1.07	1.02	1.37	3.33
OsFLS2	LOC_Os10g40990	0.95	1.16	0.81	0.62	0.52	0.35
OsNOMT	LOC_Os12g13800	1.00	20.00	1.00	1.00	93.33	50.00
OsCOMT1	LOC_Os08g06100	1.83	0.68	0.26	13.21	6.86	2.43
OsCOMT2	LOC_Os09g17560	1.00	20.00	1.00	86.67	110.00	190.00
OsCOMT3	LOC_Os08g38910	3.18	4.50	3.84	5.66	4.89	3.02
OsCOMT4	LOC_Os12g25860	1.00	1.00	1.00	1.00	1.00	1.00
OsCOMT5	LOC_Os12g25820	4.50	9.56	7.50	0.16	1.81	0.63
OsCOMT6	LOC_Os12g25490	0.61	0.09	0.05	0.61	1.11	0.89
OsCOMT7	LOC_Os12g25450	1.00	1.00	1.00	86.67	1.00	66.67
OsCOMT8	LOC_Os12g10140	1.00	1.00	1.00	1.00	1.00	1.00
OsCOMT9	LOC_Os12g09770	16.67	1.00	30.00	1.41	2.35	0.89
OsCOMT10	LOC_Os11g33300	1.00	1.00	1.00	1.00	63.33	1.00
OsCOMT11	LOC_Os11g20160	1.10	0.88	0.42	6.58	3.42	3.00
OsCOMT12	LOC_Os11g20090	4.67	3.55	4.36	1.00	1.00	1.00
OsCOMT13	LOC_Os11g20080	9.29	0.02	2.43	30.00	116.67	336.67
OsCOMT14	LOC_Os11g20040	5.36	1.18	0.64	1.00	1.00	1.00

OsCOMT15	LOC_Os11g19880	1.00	1.00	1.00	1.00	1.00	1.00
OsCOMT16	LOC_Os02g57760	0.78	0.84	0.85	0.97	1.21	0.89
OsCOMT17	LOC_Os05g43930	1.00	1.00	1.00	1.00	1.00	1.00
OsCOMT18	LOC_Os05g43940	0.75	4.25	2.92	0.02	0.64	0.02
OsF3'5'H1	LOC_Os03g25150	2.54	1.45	0.75	1.65	0.31	0.62
OsF3'5'H2	LOC_Os03g25150	1.39	1.10	0.77	3.54	2.18	1.08
OsF3'5'H3	LOC_Os02g11700	13.33	1.00	1.00	1.83	0.54	0.06
OsUGT1	LOC_Os06g09240	0.72	0.34	0.05	4.47	3.40	3.16
OsUGT2	LOC_Os06g18670	1.82	1.11	1.08	21.48	8.49	0.51
OsUGT707A5	LOC_Os07g32010	1.13	1.47	1.29	0.35	2.24	5.46
OsUGT707A2	LOC_Os07g32060	2.28	2.79	2.16	0.31	1.36	4.20
OsUGT707A4	LOC_Os07g31960	3.33	2.20	0.83	1.00	86.67	63.33
OsUGT707A3	LOC_Os07g32020	46.67	1.00	1.00	2.96	1.46	1.41
OsUGT706D1	LOC_Os01g53460	0.79	0.87	0.64	0.10	2.90	1.78
OsUGT85E2	LOC_Os02g51910	1.63	1.98	2.37	1.04	0.95	1.61
OsUGT85A8	LOC_Os02g51900	0.90	1.15	1.89	0.49	0.45	0.54
OsUGT703A2	LOC_Os01g45110	0.45	0.07	0.04	0.04	46.43	95.29
OsUGT88C3	LOC_Os01g53370	1.58	3.96	0.75	1.01	0.70	1.97
OsFNSII	LOC_Os04g01140	1.87	0.38	0.15	12.91	6.58	0.34
OsF2H	LOC_Os06g01250	0.70	1.65	0.51	2.59	1.50	0.30

Table S9 The gene expression variation factors at different ECs of SCN^-.

Gene	Roots			Shoots		
	EC_{20}	EC_{50}	EC_{75}	EC_{20}	EC_{50}	EC_{75}
OsPAL1	0.45	31.47	24.76	356.12	35.86	27.03
OsPAL2	45.96	16.90	-33.11	159.61	-6.36	23.64
OsPAL3	122.76	111.52	-24.50	410.18	-18.06	58.27
OsPAL4	120.72	24.20	-15.18	93.06	53.26	-2.47
OsPAL5	68.97	-0.65	15.12	26.63	-34.28	9.83
OsPAL6	65.75	106.06	45.78	29.16	-28.55	-25.14
OsPAL7	85.92	53.38	67.68	17.34	30.39	139.60
OsPAL8	0.00	0.00	0.00	0.00	0.00	0.00
OsPAL9	0.00	0.00	0.00	0.00	0.00	0.00
OsC4H	332.43	121.57	697.22	1233.33	-4.00	-37.63
OsC4H1	232.39	106.50	132.60	0.00	383.33	858.33
OsC4H2	50.08	80.22	-9.52	-1.67	-65.72	12.21
OsC4H3	0.00	0.00	0.00	-83.33	-99.50	-72.40
Os4CL1	41.34	136.32	36.33	-17.39	-7.20	5.26
Os4CL2	-96.67	-14.29	-95.71	38.61	-14.72	-55.58
Os4CL3	36.91	18.76	-13.03	94.75	26.40	39.51
Os4CL4	35.89	12.67	4.19	44.94	19.57	19.71
Os4CL5	59.39	36.47	82.20	89.87	17.06	175.36
OsCHS1	50.65	-6.98	179.03	267.73	29.19	-71.69
OsCHS2	-58.53	20.07	43.33	-94.00	0.00	-99.55
OsCHS5	0.00	0.00	0.00	0.00	0.00	0.00
OsCHS6	0.00	1900.00	0.00	196.15	-64.60	-71.43

OsCHS7	0.00	0.00	0.00	0.00	1900.00	0.00
OsCHS8	54.58	139.19	0.00	0.00	0.00	0.00
OsCHS9	187.50	0.00	0.00	0.00	0.00	0.00
OsCHS11	0.00	9566.67	0.00	0.00	0.00	0.00
OsCHS12	0.00	166900.00	-96.25	0.00	0.00	0.00
OsCHS13	0.00	0.00	0.00	0.00	0.00	0.00
OsCHS14	220.00	0.00	0.00	4.35	-99.79	-87.79
OsCHS15	630.00	436.36	5233.33	-68.29	100.00	73.64
OsCHS17	1566.67	0.00	0.00	0.00	0.00	0.00
OsCHS19	0.00	0.00	0.00	0.00	0.00	0.00
OsCHS20	0.00	0.00	0.00	0.00	0.00	0.00
OsCHS21	0.00	0.00	0.00	0.00	0.00	0.00
OsCHS23	0.00	0.00	0.00	0.00	0.00	0.00
OsCHS25	0.00	0.00	0.00	0.00	0.00	0.00
OsCHS26	5233.33	-97.00	0.00	52.94	-98.93	0.00
OsCHS27	0.00	-62.30	8900.00	-99.36	0.00	0.00
OsCHS28	0.00	0.00	0.00	0.00	4233.33	0.00
OsCHS28	0.00	0.00	0.00	0.00	0.00	0.00
OsCHS28	38233.33	7233.33	-95.71	8566.67	0.00	0.00
OsCHS29	0.00	0.00	0.00	0.00	0.00	0.00
OsCHS32	0.00	0.00	0.00	0.00	0.00	-98.75
OsCHI1	-9.28	9.41	25.04	154.90	9.48	-16.38
OsCHI2	6.67	-3.35	43.88	21.85	102.10	-29.60
OsCHI3	69.17	143.35	102.61	82.24	27.06	-52.78
OsCHI4	12.82	2.21	-2.40	193.45	131.71	108.41
OsCHI5	-8.01	-34.71	145.83	9.63	63.33	-8.72
OsCHI6	20.86	156.52	-13.86	212.79	59.55	-49.13
OsCHI7	101.94	50.64	97.50	177.78	43.75	-75.06
OsF3H1	7.69	200.00	2900.00	0.00	6233.33	375.00
OsF3H2	17.46	67.90	19.02	10.96	-5.72	47.90
OsF3H3	5.45	13.77	-10.08	100.00	-26.93	88.66
OsF3H4	4566.67	0.00	0.00	0.00	0.00	0.00
OsF3H5	-11.63	-3.78	-49.92	-32.71	7.64	117.35
OsF3H6	-34.01	-43.38	-50.49	-49.59	-28.73	64.47
OsF3H7	-25.93	-22.92	-88.57	-98.89	-99.09	50.00
OsF3H8	-67.46	96.00	2900.00	-62.86	-19.51	144.44
OsF3H9	49.43	84.98	51.68	-40.76	14.48	74.91
OsF3H10	38.83	-27.59	11233.33	0.00	581.25	0.00
OsF3H11	15.29	264.60	860.00	0.00	2233.33	0.00
OsF3H12	13.46	685.71	40.48	-54.60	-32.63	34.72
OsF3H13	-30.43	230.00	-38.10	-48.02	-25.97	194.87
OsF3H14	6233.33	0.00	-99.29	0.00	-96.67	-95.71
OsF3H15	5.80	-7.78	0.63	-32.81	-10.14	14.50
OsF3'H	112.48	198.53	25.00	323.04	-61.95	-62.99
OsF3'H1	0.00	0.00	0.00	0.00	0.00	0.00
OsF3'H2	36.36	166.67	414.29	-27.57	-18.73	-0.90

OsF3'H3	8.80	71.43	-29.03	0.00	0.00	0.00
OsF3'H4	21.10	208.41	125.82	-22.03	-26.71	16.98
OsF3'H5	0.00	0.00	0.00	0.00	0.00	0.00
OsF3'H6	77.27	2233.33	0.00	8566.67	0.00	0.00
OsF3'H7	48.00	600.00	0.00	0.00	880.00	-92.68
OsF3'H8	80.00	8233.33	6566.67	60.49	-33.33	-44.48
OsF3'H9	43.59	239.09	1359.52	481.89	37.90	-43.48
OsFLS1	159.02	200.00	-98.00	81.30	-71.13	121.28
OsFLS3	-2.75	57.84	33.59	-34.82	-36.36	-3.05
OsFLS2	-28.34	39.80	-5.45	-30.74	6.34	1.68
OsNOMT	0.00	1900.00	-95.71	0.00	-15.15	4900.00
OsCOMT1	76.03	99.53	50.44	668.77	-24.11	34.84
OsCOMT2	0.00	1900.00	0.00	8566.67	-28.26	18900.00
OsCOMT3	23.39	250.00	276.92	351.50	25.50	16.73
OsCOMT4	0.00	0.00	0.00	0.00	0.00	0.00
OsCOMT5	-10.00	512.00	25.00	-85.71	404.35	263.64
OsCOMT6	21.61	550.00	66.67	-48.77	59.48	0.91
OsCOMT7	-92.50	-98.13	0.00	333.33	-96.67	6566.67
OsCOMT8	0.00	0.00	0.00	0.00	0.00	0.00
OsCOMT9	1566.67	0.00	12.50	-34.97	23.06	-51.78
OsCOMT10	0.00	0.00	0.00	0.00	6233.33	0.00
OsCOMT11	-21.03	23.10	27.78	1216.67	-54.95	414.29
OsCOMT12	54.00	-7.14	311.43	0.00	0.00	0.00
OsCOMT13	306.25	-98.93	385.71	-89.16	52.17	44.29
OsCOMT14	321.43	-69.77	-65.00	0.00	0.00	0.00
OsCOMT15	-94.00	0.00	0.00	0.00	0.00	0.00
OsCOMT16	-4.15	-2.41	36.79	-22.52	49.43	-11.09
OsCOMT17	0.00	0.00	0.00	0.00	0.00	0.00
OsCOMT18	-52.63	410.00	59.09	0.00	2900.00	0.00
OsF3'5'H1	46.35	75.55	-9.20	105.81	-61.93	88.57
OsF3'5'H2	13.03	53.04	40.05	88.87	4.10	-34.26
OsF3'5'H3	1233.33	0.00	-95.71	236.00	-29.78	-91.08
OsUGT1	-10.48	960.00	2233.33	50.10	7.78	-25.28
OsUGT2	33.93	21.32	48.46	214.78	-2.75	-78.71
OsUGT707A5	-17.38	2.55	-11.46	-75.44	-45.77	109.77
OsUGT707A2	25.85	135.45	92.13	-86.08	-37.87	63.49
OsUGT707A4	53.85	200.00	257.14	0.00	0.00	6233.33
OsUGT707A3	4566.67	0.00	0.00	70.73	-35.40	58.73
OsUGT706D1	5.30	-20.83	-36.57	-94.37	27.96	-53.21
OsUGT85E2	-21.20	-23.95	-43.39	65.85	-8.61	42.75
OsUGT85A8	-28.14	-31.99	-25.26	-46.53	-25.54	6.21
OsUGT703A2	56.48	460.00	-91.43	-99.91	-45.29	249.21
OsUGT88C3	35.71	251.85	5900.00	-7.96	-60.28	-6.22
OsFNSII	135.79	106.79	290.09	403.41	55.76	-72.02
OsF2H	-35.81	387.68	27.61	29.30	14.02	-59.72
